# Case management of malaria fever in Cambodia: results from national anti-malarial outlet and household surveys

**DOI:** 10.1186/1475-2875-10-328

**Published:** 2011-10-31

**Authors:** Megan Littrell, Hellen Gatakaa, Sochea Phok, Henrietta Allen, Shunmay Yeung, Char Meng Chuor, Lek Dysoley, Duong Socheat, Angus Spiers, Chris White, Tanya Shewchuk, Desmond Chavasse, Kathryn A O'Connell

**Affiliations:** 1 Population Services International, Department of Malaria and Child Survival, P.O. Box 14355-00800, Nairobi, Kenya; 2 Population Services International Cambodia, No. 29 Street 334, P.O. Box 153, BKK1 Chamcar Mon, Phnom Penh, Kingdom of Cambodia; 3 Department of Global Health and Development, London School of Hygiene and Tropical Medicine, 15-17 Tavistock Place, London WC1H 9SH, UK; 4 National Centre of Entomology, Parasitological and Malaria control, House 372, St Monivong Vong, Boeung Keng Kang I, Chamcar Mon, Phnom Penh, Kingdom of Cambodia

**Keywords:** Malaria, Cambodia, ACT, artemisinin monotherapy, diagnosis, treatment-seeking behaviour, public sector, private sector

## Abstract

**Background:**

Continued progress towards global reduction in morbidity and mortality due to malaria requires scale-up of effective case management with artemisinin-combination therapy (ACT). The first case of artemisinin resistance in *Plasmodium falciparum *was documented in western Cambodia. Spread of artemisinin resistance would threaten recent gains in global malaria control. As such, the anti-malarial market and malaria case management practices in Cambodia have global significance.

**Methods:**

Nationally-representative household and outlet surveys were conducted in 2009 among areas in Cambodia with malaria risk. An anti-malarial audit was conducted among all public and private outlets with the potential to sell anti-malarials. Indicators on availability, price and relative volumes sold/distributed were calculated across types of anti-malarials and outlets. The household survey collected information about management of recent "malaria fevers." Case management in the public versus private sector, and anti-malarial treatment based on malaria diagnostic testing were examined.

**Results:**

Most public outlets (85%) and nearly half of private pharmacies, clinics and drug stores stock ACT. Oral artemisinin monotherapy was found in pharmacies/clinics (9%), drug stores (14%), mobile providers (4%) and grocery stores (2%). Among total anti-malarial volumes sold/distributed nationally, 6% are artemisinin monotherapies and 72% are ACT. Only 45% of people with recent "malaria fever" reportedly receive a diagnostic test, and the most common treatment acquired is a drug cocktail containing no identifiable anti-malarial. A self-reported positive diagnostic test, particularly when received in the public sector, improves likelihood of receiving anti-malarial treatment. Nonetheless, anti-malarial treatment of reportedly positive cases is low among people who seek treatment exclusively in the public (61%) and private (42%) sectors.

**Conclusions:**

While data on the anti-malarial market shows favourable progress towards replacing artemisinin monotherapies with ACT, the widespread use of drug cocktails to treat malaria is a barrier to effective case management. Significant achievements have been made in availability of diagnostic testing and effective treatment in the public and private sectors. However, interventions to improve case management are urgently required, particularly in the private sector. Evidence-based interventions that target provider and consumer behaviour are needed to support uptake of diagnostic testing and treatment with full-course first-line anti-malarials.

## Background

Financing for malaria control has increased substantially over the last decade, facilitating significant progress towards international targets for prevention and treatment. Increased coverage of at-risk populations with vector control as well as effective case management with artemisinin combination therapy (ACT) is contributing to substantial reductions in malaria cases and deaths [1]. The spread of artemisinin resistance in *Plasmodium falciparum *malaria parasites would threaten recent malaria control progress across endemic countries. Alternative anti-malarial medicines with equivalent levels of efficacy are not expected to become available for at least seven to eight years. *P. falciparum *resistance to artemisinin derivatives has already begun to emerge; the first case was confirmed in Cambodia, near the Thai border (Pailin province) in 2009 [2,3].

Factors believed to be contributing to emerging drug resistance in Cambodia include the unregulated sale of artemisinin monotherapies for over 40 years; limited access to ACT; co-blistered ACT which is not co-formulated (facilitating continued use of artemisinin monotherapy); and ubiquitous counterfeit and substandard drugs [2]. Cambodia's resistance containment programme consists of a number of interventions to facilitate early diagnosis and appropriate treatment of malaria. These include a ban on the sale of artemisinin monotherapies introduced in 2009; ongoing efforts to strengthen capacity for drug quality monitoring; a new bureau for policing private drug sellers; and active efforts to close unlicensed pharmacies [2,4,5]. To facilitate diagnosis and treatment with national first-line drugs, these services are made available at community level through a village malaria worker (VMW) programme implemented in remote provinces [2,6]. Co-blistered artesunate + mefloquine (ASMQ) is the first-line treatment for *P. falciparum *and chloroquine is currently the first-line treatment for *P. vivax *infections. In late 2008, the first-line drug in districts with confirmed multidrug resistance was changed to dihydroartemisinin + piperaquine (DHA+PPQ); public outlets began stocking the drug in 2009 [2].

Other national malaria control efforts include provision of highly subsidized rapid diagnostic testing (RDT) and ACT treatment in the private sector. The international NGO, Population Services International (PSI) has led private sector malaria treatment in Cambodia since 2003. Private sector ASMQ is sold under the brand name Malarine and accounts for 75% of the ASMQ distributed nationally. Rapid diagnostic test kits (RDT) are sold under the brand name Malacheck. At the time of data collection for this study, Malacheck tested for *P. falciparum *infections. In 2010, the diagnostic kit changed to test for both *P. falciparum *and *Plasmodium vivax *infections. Malarine and Malacheck are sold in over 1,700 outlets, including private hospitals, clinics, pharmacies, mobile providers and drug stores [4]. In the public sector, ASMQ is available under the name A+M.

Malaria control in Cambodia is of high significance globally because of the risk of *P. falciparum *resistance to artemisinin, which could develop and spread to sub-Saharan Africa, crippling malaria control programmes in high-burden countries. Despite its importance, national level data on the anti-malarial market and on household treatment-seeking behaviour are limited. Prior to this study, national data collection efforts were undertaken by the national malaria control programme (National Centre of Entomology, Parasitological and Malaria control, CNM) in 2007 [7]. Given a dynamic policy and programming environment, the need for national-level data that can continually inform targeted malaria control and drug resistance containment efforts is important.

This study uses data collected in 2009 as part of the *ACTwatch *research programme [8]. *ACTwatch *is designed to provide a comprehensive picture of the anti-malarial market to inform national and international drug policy evolution [9]. Cambodia is one of seven *ACTwatch *study countries. Nationally-representative outlet and household surveys were conducted to examine the supply and demand side of the anti-malarial market. Data from this study complement existing data on malaria treatment in Cambodia by providing a comprehensive picture of the anti-malarial market, including the availability, price and volumes of various anti-malarials moving through both the public and the private sector. This study also presents national data on household treatment-seeking behaviour for "malaria fever." Results can be used as part of monitoring access to and demand for effective combination treatment, as well as availability and consumption of artemisinin monotherapies. Information on consumer and provider behaviour can inform communications aimed at changing behaviour in the context of increased access to diagnostic testing and ACT.

## Methods

This study uses data from two nationally-representative cross-sectional surveys. A survey of outlets with the potential to sell or distribute anti-malarials was conducted in June through July, 2009. A household survey was conducted in October through November, 2009. Data collection coincided with the rainy season during which malaria transmission occurs; this season generally begins in late May and ends in December.

### Design and sampling

Both outlet and household survey sampling began with drawing a sample of 38 administrative clusters (health centre catchment areas) selected with probability proportional to size (PPS) from stratified lists of the 255 clusters in malaria endemic areas of Cambodia. Equal allocation stratification was utilized to allow for separate estimates across two malaria-endemic strata: areas with confirmed or suspected multidrug resistance and areas without confirmed/suspected multidrug resistance. A census of all outlets in these clusters was completed. To allow for comparisons between outlet types, public health facilities were also sampled from a larger administrative "booster" area (district) in which the selected cluster was located. A total of 7,518 outlets were visited. In the public sector, these included public health facilities (government referral hospitals, health centres and posts, N = 211) and village malaria workers (VMW, N = 203). VMWs are stocked with RDTs and anti-malarials and provide testing and treatment to the community free of charge. Government health facility staff support VMWs with initial training, monthly supervision and stock. Private sector outlets include nationally registered pharmacies/clinics (pharmacies, clinical pharmacies, cabinets, and private clinics, N = 152); informal drug stores (N = 164); grocery stores (shops located in urban areas, N = 1,170); village shops (shops located in rural areas, N = 5,144); and mobile providers (N = 383). Mobile providers are found predominately in rural areas and serve the communities in which they reside. They typically possess medical training and are often currently or previously employed by a public or private sector facility. Mobile providers interviewed in the outlet survey provided answers with respect to their drug stock and practices operating outside the realm of any other outlet that they are affiliated with. However, some mobile providers reportedly offer diagnostic testing using microscopy as part of their practice, in which case they take blood slides from patients in their mobile provider practice and use a facility laboratory for diagnosis.

Within the 38 clusters selected with PPS as first stage, a second stage of selection was used to select six census enumeration areas per cluster with PPS. Within each census enumeration area, 100 households were randomly selected for screening. The number of households in each census enumeration area was divided by 100 to derive a skip interval for random systematic sampling of households. A total of N = 22,317 households were asked a series of screening questions concerning fevers occurring among household members in the previous two weeks. While N = 14,306 recent fevers (*krun kdao*) were identified, interviews were conducted only among the N = 1,617 people with recent self-reported "malaria fever" (*krun chanh/krun gnak*). Most "malaria fevers" included in this study had occurred among men (62%) and people age 15 and above (71%).

### Materials

The core component of the outlet survey instrument is an exhaustive audit of all anti-malarials in stock at the time of the survey. For each anti-malarial in stock, identifying information including brand, generic and manufacturer names and drug formulation and strength was collected. Provider reports on unit cost and amount sold or distributed within the last week was also recorded. The survey additionally measured availability and price of microscopic and rapid diagnostic blood testing. Basic outlet and provider characteristics were collected and provider knowledge surrounding first-line treatment was assessed.

The household survey instrument collected detailed information on treatment-seeking behaviour, including type, timing, source, and price paid for diagnostic testing and drugs acquired for fever. Respondent recall and recognition of the type of treatment acquired was aided by the use of a comprehensive anti-malarial field guide with photographs and brand names of common anti-malarials available in public and private sector outlets. This field manual included pictures of anti-malarial tablets that are not part of pre-packaged therapies. Where respondents reported use of a combination of drugs - or a multi-drug cocktail, the anti-malarial field guide was used to help the respondent identify anti-malarials contained within the cocktail, where applicable. Non-anti-malarial cocktail contents (e.g. vitamins, antipyretics) were identified by respondent recall, unaided by a pictorial guide.

The household survey was administered to people age 15 and above with "malaria fever," and to children's primary caregiver where the person with fever was below age 15. In addition to fever management questions, respondents were asked a series of questions assessing their attitudes and knowledge regarding malaria diagnosis and treatment. A household questionnaire module, modelled after the Demographic and Health Survey (DHS) [10], collected information on household characteristics and household assets to be used in assessment of relative socioeconomic status.

### Training and fieldwork

Data collection teams received a six-day training focused on administration of the questionnaire and sampling procedures. The outlet survey was conducted in June and July, 2009. Household survey data were collected from people with "malaria fever" during the two weeks preceding the survey (or their primary caregiver if under the age of 15) during the months of September, October and November, 2009, falling within the peak malaria transmission season. All questionnaires were reviewed by the team supervisor and spot checks were conducted for at least 20% of all outlets and households. Microsoft Access (^©^Microsoft Corporation, Seattle, WA, USA) was used for double data entry and validation. All research activities operate under ethical approval granted by the Cambodian national ethics review board.

### Measures

Anti-malarials identified during the outlet drug audit were classified according to information on drug formulation, contents and strengths with supporting information including brand or generic name and manufacturer. All outlets visited were classified as having at least one anti-malarial in stock at the time of the survey or not. Cotrimoxazole and medicines intended solely for malaria chemoprophylaxis were not categorized as anti-malarials. Among outlets stocking anti-malarials, variables were created to indicate stocks by type, including the broad category of ACT, as well as specific categories including all ASMQ, private sector ASMQ sold as Malarine, public sector ASMQ distributed as A+M, oral artemisinin monotherapy, and non-artemisinin monotherapy. Availability of diagnostic testing services was assessed among outlets stocking anti-malarial(s) on the day of the interview, or that reportedly stocked anti-malarial(s) within the past three months. Outlets were categorized according to availability of rapid diagnostic tests (in stock on the day of the interview) and microscopic blood testing (availability of services). Outlet possession of laboratory equipment in working order was not assessed. Provider reports on the availability of microscopic blood testing services - which could entail taking patient blood slides to another site for testing (particularly relevant in the case of mobile providers) - were used to classify availability of microscopic blood testing.

To calculate volumes of anti-malarials reportedly sold or distributed in the week preceding the survey as well as drug prices, drug courses were standardized using adult equivalent treatment doses (AETD). One AETD was defined as the amount of the drug needed for a full course treatment based on guidelines from the WHO where available. Where unavailable from the WHO, peer-reviewed literature was consulted, and if necessary, manufacturer guidelines were utilized. Provider reports on drug prices per unit (e.g. tablet) and amount of the drug sold or distributed during the week preceding the survey were used to calculate volumes and price according to type of anti-malarial: Malarine, A+M, other ACT, oral artemisinin monotherapy, non-oral artemisinin monotherapy and non-artemisinin monotherapy. The volume of each drug is therefore the number of AETDs that were reportedly sold/distributed during the week preceding the survey. The price of each drug was calculated for one AETD. Audited anti-malarials with missing data required to calculate AETD - specifically drug strength - were excluded from volumes and price estimation.

Household survey indicators of treatment-seeking behaviour and treatment of fever were constructed from respondent reports on sources where treatment was sought; whether or not the person with fever received a diagnostic test; self-reported diagnostic test result; source of diagnostic test and treatment(s); type of treatments acquired (brand names); and timing of treatments. Brand names were used to categorize drugs according to generic anti-malarial types (e.g. chloroquine, quinine, ASMQ). These were then further classified as ACT, artemisinin monotherapy, or non-artemisinin monotherapy. Indicators were calculated using the three classes of anti-malarial above, as well as an overall category for *any anti-malarial*. These anti-malarial drug categories do not include anti-malarials that were taken as part of a drug cocktail. A separate category was created to indicate cocktail treatments that contained an identifiable anti-malarial. Another drug category was created for people who received a cocktail of drugs that did not contain any identifiable anti-malarial.

Sources for diagnosis and treatment were categorized as either public or private sector. Public health facilities, non-profit health facilities and village malaria workers (VMWs) were classified as public sector. The private sector encompasses outlets with providers who have formal training (pharmacies, clinical pharmacies, cabinets, private clinics, mobile providers) as well as providers who do not generally have formal training (drug stores, grocery stores, village shops). Sector in which a person with "malaria fever" sought treatment was defined as public only, private only, or a mix of public and private outlets.

Respondent knowledge and beliefs about the first-line treatment, ASMQ, was assessed through two items. One dichotomous variable indicates whether or not the respondent names ASMQ (generic or a brand name) as an anti-malarial drug. A second measure captured respondent beliefs on the most effective treatment for adults with malaria through an open-ended question that was re-coded into categories including ASMQ and other ACT.

Socioeconomic status was assessed at the household level relative to other household respondents, using measures of housing, water, sanitation and household asset items modelled after the DHS. Wealth index items were assigned a weight through principal components analysis and standardized in relation to a standard normal distribution. Each respondent was categorized according to their household's socioeconomic score, and recorded into one of five wealth quintiles, ranging from lowest/poorest to highest/least poor [11].

### Data analysis

Frequencies were tabulated for availability of anti-malarials and diagnostic testing by outlet type. Volumes of anti-malarials sold or distributed during the previous week was calculated by considering the fraction of overall volumes accounted for by the number of AETDs sold/distributed per anti-malarial drug type (public sector ASMQ - A+M, private sector ASMQ - Malarine, other forms of ACT (dihydroartemisinin+piperaquine - DHA+PPQ, or artemisinin+piperaquine - A+PPQ), oral artemisinin monotherapy, non-oral artemisinin monotherapy, non-artemisinin monotherapy). Frequencies of AETDs reportedly distributed for free in the public sector were calculated across drug types: the most popular non-artemisinin monotherapy, chloroquine; oral artemisinin monotherapy; Malarine; A+M; and other forms of ACT. Median price and interquartile range of AETDs sold in the private sector were calculated across these drug types. Median price was also calculated for RDTs and microscopic blood testing available in the private sector.

At the household level, frequencies were tabulated for source of initial treatment, sector in which treatment was sought (for those who sought treatment outside of the home), and source of diagnostic testing and treatments. Frequencies were also tabulated for number of treatments acquired for one episode of "malaria fever," and type of treatments acquired. Logistic regression was used to test for an association between treatment-seeking sector (public only, private only, public and private) and: 1) diagnostic testing among all people with fever; 2) anti-malarial treatment of self-reported positive cases, self-reported negative cases, and undiagnosed "malaria fevers" for which treatment was sought in the public and/or private sector. Logistic regression was also used to test for an association between household socioeconomic status and diagnostic testing among all people with "malaria fever." Odds ratios and 95% confidence intervals are reported.

Household data were weighted to account for difference in the probability of being selected in the different strata. Standard error estimation accounted for clustering at the health centre catchment area and enumeration area levels. Given the use of a census approach, sampling weights were applied to outlet survey data based on the inverse probability of selection to account for differences in strata and cluster sizes and the oversampling of booster outlet types. Outlet distribution was assumed to be proportional to population size. Stata 11.0 (^©^Stata Corp, College Station, TX, USA) was used for all analyses.

## Results

### Outlet survey

Most public health facilities (PHF, 85%) and village malaria workers (VMW, 91%) stock anti-malarials. Among private sector outlets, percentage of outlets stocking anti-malarials is highest among pharmacies/clinics (54%) and drug stores (52%), and relatively low among grocery stores (8%) and village shops (2%). About one-third (32%) of mobile providers stock anti-malarial drugs. Most public sector outlets stock ACT (PHF, 84%; VMW, 86%). While 49% of pharmacies/clinics and 45% of drug stores had ACT at the time of the interview, ACT is less commonly available among mobile providers (20%), grocery stores (4%) and village shops (1%) (Table 1).

**Table 1:** Percentage of outlets with stock of diagnostic testing and anti-malarials on the day of the interview

	Public sector			Private sector					
	
	Public Health Facility	Village malaria worker	Public sector total	Pharmacy/Clinic	Drug Store	Grocery Store	Village shop	Mobile Provider	Private sector total
**All outlets, % stocking:**	**N = 211**	**N = 203**	**N = 414**	**N = 152**	**N = 164**	**N = 1,170**	**N = 5,144**	**N = 383**	**N = 7,013**

									
Any anti-malarial	85.0	91.1	88.4	53.7	51.9	7.8	2.4	32.4	7.2
Any ACT	83.6	86.3	85.1	49.0	45.4	4.0	0.9	19.9	4.5
Any ASMQ^1^	82.9	80.8	81.7	44.2	43.6	3.8	0.8	18.2	4.2
A+M^1^	81.3	80.8	81.0	5.6	7.8	0.1	0.1	5.0	0.7
Malarine^1^	3.5	0.3	1.7	40.6	38.4	3.8	0.7	16.4	3.8
Other ACT^2^	2.8	6.5	4.8	13.7	5.5	0.3	0.2	2.6	0.8
Oral artemisinin									
monotherapy	0.0	3.7	2.1	9.4	13.8	2.0	0.5	4.2	1.5
Non-artemisinin									
monotherapy	40.7	55.1	48.8	14.9	16.1	4.1	1.5	14.1	3.3

Outlets stocking any anti-malarial in the past 3 months, % stocking:	N = 183	N = 192	N = 375	N = 96	N = 103	N = 91	N = 151	N = 202	N = 643

									
Any diagnostic test	83.3	76.2	79.1	73.6	62.8	35.8	11.8	74.2	51.9
RDT	74.0	76.2	75.3	57.9	38.5	33.6	8.7	41.5	34.2
Microscopy	36.9	2.1	16.7	37.9	32.3	3.6	3.7	45.2	26.1

1 Private sector ASMQ sold under the name Malarine and public sector ASMQ distributed under the name A+M

2 Other ACTs are DHQ+PPQ or A+PPQ

Most public sector outlets stock ASMQ (PHF, 83%; VMW, 81%). Fewer than half of all pharmacies/clinics (44%) and drug stores (44%) have ASMQ in stock. Pharmacies, clinics and drug stores tend to stock the private sector ASMQ, Malarine, and stocking of the public sector A+M is uncommon (pharmacies/clinics, 6%; drug stores, 8%; mobile providers, 5%). These results suggest minimal leakage of public sector ACT to the private sector. The category "other ACT" largely refers to dihydroartemisin+piperaquine (DHA+PPQ), although artemisinin+piperaquine (A+PPQ) is also available to some extent. Few public sector outlets are stocking other forms of ACT (PHF, 3%; VMW, 7%). However stocking rates are relatively higher in the private sector: 14% of pharmacies/clinics; 6% of drug stores; and 3% of mobile providers (Table 1). Consistent with national treatment policy stipulating the use of DHA+PPQ for the first-line ACT in artemisinin resistance Containment Zone 1 (a recent shift from ASMQ at the time of the survey), all public sector and most private sector "other ACT" are found in areas of Cambodia with suspected or confirmed drug resistance (Zones 1 and 2, data not shown). Oral artemisinin monotherapies are not found at PHF. In the private sector, 9% of clinics/pharmacies, 14% of drug stores, 4% of mobile providers, and 2% of grocery stores are carrying oral artemisinin monotherapies (Table 1).

Non-artemisinin monotherapies found in the public and private sector are most commonly chloroquine treatments (the first-line for *P. vivax*), although quinine (the second-line drug for *P. falciparum*) is also common to some extent. Non-artemisinin monotherapy is available among 41% of PHF and 55% of VMWs. In the private sector, stocking rates of non-artemisinin monotherapies are low: 15% of pharmacies/clinics; 16% of drug stores; 14% of mobile providers; and 4% of grocery stores (Table 1).

Most anti-malarial-stocking public sector outlets have diagnostic testing available (PHF, 83%; VMW, 76%), with RDTs (75%) more commonly available in the public sector as compared with microscopic testing (17%). Among anti-malarial-stocking private sector outlets, diagnostic testing is more commonly available within pharmacies/clinics (74%), drug stores (63%) and mobile providers (74%), and less commonly available among grocery stores (36%) and village shops (12%). RDTs are more commonly available in the private sector as compared with microscopy with the exception of mobile providers; 42% have RDTs in stock and 45% report providing microscopic testing services (Table 1).

**Figure 1:**
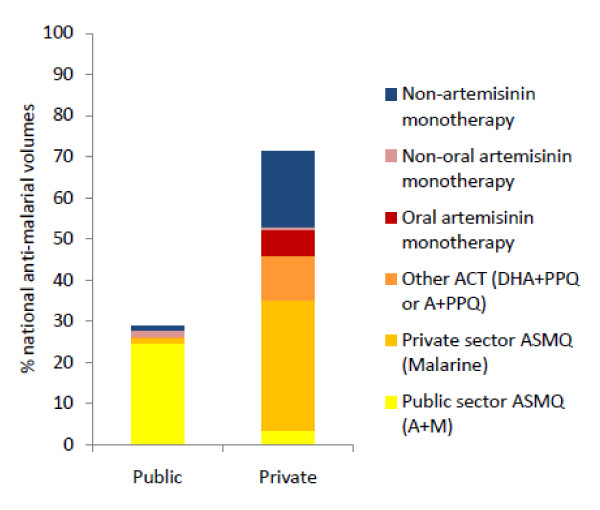
**Volumes of anti-malarial medicines sold/distributed in the past week**.

Figure 1 shows the relative volumes of anti-malarial medicines that were sold or distributed by all outlets in the past week. The public sector accounts for 29% of the total market share, and this is largely comprised of ASMQ; public sector ASMQ accounts for 26% of the total volumes. The private sector accounts for 71% of the total market share, and this is accounted for primarily by ASMQ (32% Malarine, 3% A+M) and non-artemisinin monotherapy (19%). Across sectors, most of the anti-malarials sold/distributed are ACT (72%), including Malarine (33%), A+M (28%), and other ACT - primarily DHA+PPQ (11%). Non-artemisinin monotherapies - primarily chloroquine - account for 19% of the total volumes, and oral artemisinin monotherapies account for 6% of total volumes (Figure 1).

Anti-malarials and diagnostic testing are reportedly distributed free of charge in the public sector in Cambodia, although service fees typically apply. In the private sector, the median price charged for an adult equivalent treatment dose (AETD) of the most popular non-artemisinin monotherapy is just $0.27 (IQR = $0.23-$0.46), compared with $1.18 (IQR = 0.82-1.65) for private sector Malarine and $2.12 (IQR = $1.69-$2.17) for other types of ACT. An oral artemisinin monotherapy AETD is reportedly distributed for a median price of $3.62 (IQR = $3.01-4.52). RDTs are sold for a median price of $0.35 (IQR = $0.24-$0.47), and the median price for microscopic testing in the private sector is $0.71 (IQR = $0.59-$1.18) (Table 2).

**Table 2:** Median private sector price of anti-malarials and diagnostic tests

	N	Median price (IQR)
Chloroquine^1^	58	$0.27 ($0.23, $0.46)
Oral artemisinin monotherapy	121	$3.62 ($3.01, $4.52)
Malarine (ASMQ)	338	$1.18 ($0.82, $1.65)
Other ACTs^2^	62	$2.12 ($1.69, $2.17)

RDT	208	$0.35 ($0.24, $0.47)
Microscopy	174	$0.71 ($0.59, $1.18)

1 Chloroquine is the most popular non-artemisinin monotherapy on the market

2 Other ACTs are DHA+PPQ or A+PPQ

When asked to name the recommended first-line treatment for uncomplicated *P. falciparum *malaria, 76% of all providers in the public and private sector stated the correct response of ASMQ. Knowledge is highest among providers in PHF and VMWs (96%) and in private pharmacies/clinics (88%), and lower among providers in drug stores (74%), mobile providers (76%), grocery stores (64%) and village shops (47%) (Table 3).

**Table 3:** Percentage of providers in anti-malarial-stocking outlets who correctly state the recommended first-line treatment for uncomplicated malaria

	N	%
Public health facility & village malaria worker	372	96.1
Pharmacy/clinic	96	87.8
Drug store	101	73.7
Grocery store	91	64.4
Village shop	152	47.2
Mobile provider	202	76.2

### Household survey

**Figure 2:**
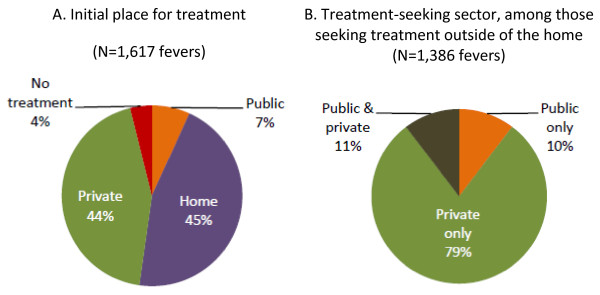
**Initial place treatment was sought and sector sought for treatment outside of the home**.

Among people with recent "malaria fever," nearly half (45%) first do something at home to treat the fever; 44% seek treatment in the private sector; 7% seek treatment in the public sector; and 4% have not yet done anything to treat the fever at the time of the interview (Figure 2). Among those people who seek treatment outside of the home at some point during the fever episode (86%), most go to just one outlet type (75%, Table 4). Among those seeking treatment outside of the home, 79% seek treatment exclusively in the private sector; 10% exclusively in the public sector; and 11% in both the public and private sectors (Figure 2).

**Table 4:** Percentage of people with "malaria fever" who seek treatment outside of the home, and number of outlet types visited

	N	%
Seek treatment outside of the home	1,617	86.1
Number of outlet types visited for treatment, among those who seek treatment:	1,386	
One	............	74.7
Two	............	22.8
Three	............	2.5

Nearly half (45%) of all people with recent "malaria fever" reportedly receive a diagnostic test. Most of these tests are RDTs (60%), while 31% are microscopy and 10% of respondents are unsure about the test type. A large percentage of those tested, 87%, report receiving a positive diagnosis; 11% reportedly receive a negative result and 1% do not know the test result (Table 5).

**Table 5:** Diagnostic testing among people with "malaria fever"

	N	%
Received a diagnostic test	1,551	44.9
Type of test, among those tested	698	
RDT	............	59.5
Microscopy	............	30.5
Don't know	............	10.0
Self-reported test result, among those tested	698	
Positive	............	87.4
Negative	............	11.4
Don't know	............	1.2

People with "malaria fever" who seek treatment exclusively in the public sector are significantly more likely to receive a diagnostic test than those who seek treatment solely in the private sector (OR = 4.52, 95% CI = 2.27-9.00). Diagnostic testing is high among those who seek treatment in the public sector only (77%) or in the public and private sector (70%) as compared with those who seek treatment in the private sector only (42%). Compared to people living in the poorest households, those in the least poor households are significantly more likely to receive a malaria diagnostic test (OR = 1.64, 95% CI = 1.05-2.55) (Table 6).

**Table 6:** Malaria diagnostic testing across treatment-seeking sector and across relative household wealth

	N	Received a diagnostic test	
		**%**	**OR (95% CI)**

Treatment-seeking sector			
Private only	1,094	42.2	1.00
Public only	145	76.8	4.52 (2.27-9.00)***
Public & private	147	69.8	3.15 (1.81-5.48)***
			
Household wealth			
Lowest	339	41.9	1.00
Low	309	42.1	1.01 (0.71-1.44)
Middle	271	49.7	1.37 (0.99-1.90)
High	318	40.0	0.85 (0.63-1.16)
Highest	314	54.1	1.64 (1.05-2.55)*

* p < 0.05 **p < 0.01 ***p < 0.001

Table 7 examines anti-malarial treatment in the public, private and public/private sectors across diagnostic test result. Among people who report a positive diagnostic test result, those who seek treatment exclusively in the public sector are significantly more likely to receive anti-malarial treatment as compared with those who seek treatment solely in the private sector (OR = 2.15, 95% CI = 1.39-3.33). Nonetheless, only 61% of people reporting a positive test result and treated in the public sector report receiving an anti-malarial, as compared with 54% of reportedly positive cases seen in the public and private sector, and 42% of cases seen exclusively in the private sector (Table 7).

**Table 7:** Anti-malarial treatment acquired for "malaria fever" across treatment-seeking sector according to self- reported malaria diagnostic test result

	N	Received any anti-malarial	
		**%**	**OR (95% CI)**

Reported positive test			
Private only	409	41.8	1.00
Public only	99	60.7	2.15 (1.39-3.33)**
Public & private	80	53.9	1.63 (0.89-2.98)
All positive cases	588	46.6	---
			
Reported negative test:			
Private only	48	13.5	1.00
Public only	11	0.0	---
Public & private	23	12.6	0.92 (0.17-5.20)
All negative cases	82	11.4	---
			
No diagnostic test:			
Private only	630	11.4	1.00
Public only	34	25.3	2.64 (0.66-10.59)
Public & private	44	16.7	1.57 (0.58-4.24)
All cases without testing	708	12.3	---

* p < 0.05 **p < 0.01 ***p < 0.001

While none of the reportedly negative cases treated solely in the public sector report receiving an anti-malarial, 14% of those treated in the private sector and 13% of those treated in the public and private sectors reportedly receive anti-malarial treatment. Among those people who did not receive a diagnostic test but sought treatment outside of the home, there is no significant difference in anti-malarial treatment across treatment-seeking sector (private only, 11%; public only, 25%; public and private, 17%) (Table 7).

**Figure 3:**
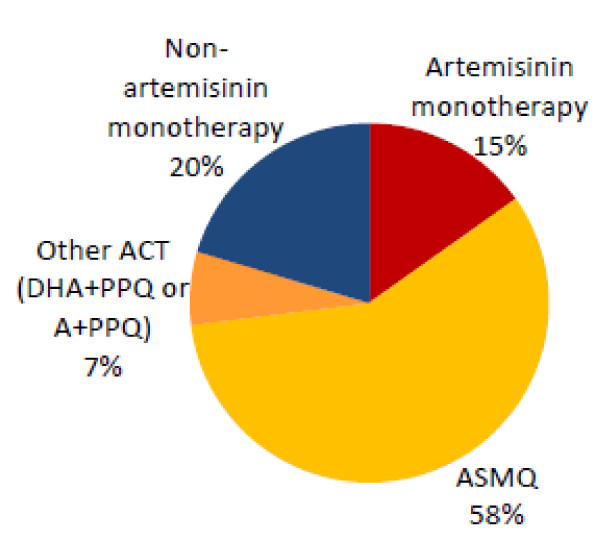
**Type of anti-malarials reportedly acquired for "malaria fever" (N = 426 anti-malarials)**.

About half of all people with "malaria fever" report taking one anti-malarial or cocktail treatment (54%). Another 21% report taking two anti-malarial and/or cocktail treatments, and 7% report taking three or more of these treatments. Eighteen percent had not yet taken any anti-malarial or cocktail treatment for the fever at the time of the survey. Most people with "malaria fever" report receiving a cocktail of drugs; 47% receive a cocktail with no identifiable anti-malarial, and another 11% receive a cocktail with anti-malarial(s) included in the mix. An anti-malarial treatment, without cocktail treatment, was reportedly received by just 15% of people with "malaria fever." Anti-malarial plus cocktail treatment was received by 10% of people with fever (Table 8). Most of the anti-malarials acquired for treatment of "malaria fever" are ACT (58% ASMQ, 7% other types of ACT), however 15% are artemisinin monotherapies and 20% non-artemisinin monotherapies - primarily chloroquine (Figure 3).

**Table 8:** Treatment acquired among people with "malaria fever"

	% (N = 1,617 fevers)
Number of treatments acquired (cocktails and anti-malarials)	
Zero	17.7
One	53.8
Two	21.4
Three or more	7.16
Type of treatment received	
Anti-malarial(s) only	14.5
Anti-malarial(s) plus cocktail(s)	9.9
Cocktail(s) containing an anti-malarial	10.6
Cocktail(s) with no identifiable anti-malarial only	47.4

**Figure 4:**
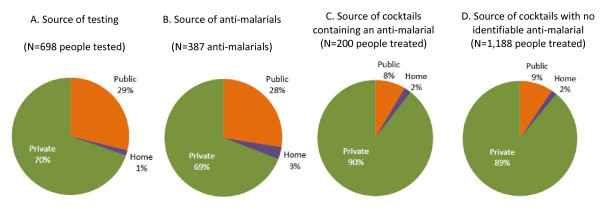
**Source of diagnostic testing, anti-malarial treatments, and cocktail treatments**.

The private sector is the most common source reported for diagnostic testing (70% of people tested) and anti-malarial treatment (69% of anti-malarial treatments acquired). Cocktail treatments are acquired primarily from the private sector (90% of cocktails containing an anti-malarial, 89% of cocktails with no identifiable anti-malarial) (Figure 4)

## Discussion

### Availability, volumes and use of anti-malarials and diagnostic testing

Outlet and household survey results show that the sale of artemisinin monotherapies is still a problem in Cambodia, however use is limited. Oral artemisinin monotherapies account for 6% of total anti-malarial volumes sold/distributed according to outlet survey results, and 15% of anti-malarial treatments reportedly consumed by individuals with "malaria fever" according to household survey results. Availability is limited primarily to the private sector and stocking rates are relatively low; oral artemisinin monotherapy is found in about one in ten pharmacies, clinics and drug stores and among 4% of mobile providers and 2% of grocery stores. Median price of a full course of treatment for an adult (i.e. seven days) in the private sector ($3.62) is more than 13 times the price for a full course of chloroquine ($0.27), and three times the price of private sector ASMQ, Malarine ($1.18). However, it is important to note that in practice, the limited use of artemisinin monotherapy is likely in the form of less expensive incomplete doses rather than full-course treatments. The cost of an adult equivalent treatment dose reported in this study does not reflect what consumers likely pay when they obtain oral artemisinin monotherapy, and therefore cost may not be a significant barrier to use. Previous studies in Cambodia have documented widespread availability and use of artemisinin monotherapies [12,13]. Recent efforts to control the use of monotherapies have included a ban on artemisinin monotherapies and active enforcement of this ban [2]. Results from this survey show that in 2009, progress was being made towards eliminating their use, but continued efforts are still needed.

ACT sales and distribution are high in Cambodia, and the majority of ACT formulations sold/distributed are the first-line drug, ASMQ. ACT account for 72% of anti-malarial volumes sold/distributed, and for 65% of anti-malarial treatments reportedly acquired for "malaria fever." The use of non-artemisinin monotherapies, largely chloroquine, accounts for 19% of volumes and 20% of treatments reportedly acquired for fever. At the time of these surveys, chloroquine was the first-line treatment for *P. vivax *infections, and therefore availability and use of this drug cannot necessarily be considered unfavourable. The relatively high use of ACT appears to be in part supported by favourable availability. Most public health facilities (84%) and VMWs (86%) provide ACT, and nearly half of private pharmacies, clinics and drug stores stock ACT. The stocking rates of non-artemisinin monotherapies (primarily chloroquine) are substantially lower: about half of public health facilities and only about 15% of pharmacies, clinics and drug stores stock these drugs. Favourable availability of ACT in the private sector is supported by the national private sector subsidy for the sale of ASMQ marketed as Malarine [4]. This mature subsidy programme was in operation at scale for five years prior to this study, and results point to the success of the programme. Results from this study show that one-third of all anti-malarials reportedly sold/distributed in Cambodia are Malarine treatments. Favourable availability of ASMQ in the public sector - including public health facilities and among community-based village malaria workers - illustrates the success of national efforts to ensure availability of effective treatment in the public health system.

In line with WHO guidelines [14], national policy in Cambodia specifies that all people with suspected malaria should first receive a diagnostic test [1]. Findings from this study show that only 45% of people with "malaria fever" in Cambodia receive a diagnostic test. The outlet survey results show that diagnostic testing is often available within anti-malarial-stocking facilities, and that RDTs are more commonly available as compared with microscopic testing. Approximately eight in ten public outlets, three-quarters of anti-malarial-stocking pharmacies and clinics, and well over half of anti-malarial-stocking drug stores (63%) provide diagnostic testing. Higher levels of availability in public health facilities and with VMWs could be contributing to higher frequency of diagnostic testing among people seeking treatment in the public sector (77% of fevers tested) as compared with those who are treated exclusively in the private sector (42%). Another barrier to diagnostic testing is cost: household survey results find that diagnostic testing is significantly higher among people from the relatively least poor households as compared to the very poorest. RDTs are available in the public sector free of charge, but with a fee for service, and providers in the private sector report a median RDT price of $0.35. Although RDTs are already heavily subsidized [4], the added cost of testing may still be prohibitive for the very poor.

Price of diagnostic testing and treatment in the *private *sector is particularly relevant in Cambodia because it is much more frequently accessed for fever diagnosis and treatment. Household survey results show that most people eventually seek treatment outside of the home for "malaria fever," (86%), and that 79% of these treatment-seekers seek care exclusively in the private sector. An additional 11% seek care in both the public and private sectors. About 70% of diagnostic tests and anti-malarial treatments are acquired in the private sector, which triangulates with outlet survey results showing that 71% of anti-malarial volumes are sold/distributed through the private sector. Similar findings have been reported elsewhere [7,12], and point to the importance of considering the private sector in efforts to improve effective fever management and eliminate the use of artemisinin monotherapies. While significant achievements have been realized in making RDTs and ACT available in private outlets - particularly pharmacies/clinics and drug stores, results from this study suggest that private sector improvements should include further expansion of private sector availability as well as appropriate sale/use of RDTs and full-course anti-malarial treatments. Where sub-optimal availability of testing and treatment remains in private sector outlets, it is essential that private providers refer clients with "malaria fever" to public or private outlets where testing and appropriate treatment are available. Interventions to strengthen linkages between private and public providers are important in Cambodia where people frequently seek treatment in the private sector. CNM and its partners are currently pursuing a public-private mix (PPM) approach that entails training, incentive systems and a certification program for private providers [15].

### Cocktail treatments

ACT availability and price are favourable, particularly compared to artemisinin monotherapies. Further, the proportion of anti-malarials that are sold/distributed and reportedly consumed in Cambodia are overwhelmingly ACT. However, household study results show that the state of "malaria fever" management in Cambodia is far from ideal. Findings from this national study confirm results from smaller quantitative [12,13,16] and qualitative [17] studies on the widespread use of cocktail treatments in Cambodia's private sector. Cocktail treatment for suspected malaria in Cambodia typically consists of a small plastic bag containing one or more tablets of various drugs including antipyretics, vitamins, anti-malarials, antihistamines and antibiotics [12,13]. Cocktail treatment presents a significant barrier to full-course treatment. Those seeking treatment may not be provided with the full course, either due to provider practices or cost of treatment. Even if they happen to receive a full course of anti-malarial treatment as part of a cocktail, consumers may not receive adequate information on the cocktail contents and instructions for complying with the treatment regimen.

Among all people with "malaria fever," only 15% report receiving an anti-malarial treatment only (without acquiring cocktail treatment). Another 10% receive an anti-malarial as well as cocktail treatment. Higher levels of anti-malarial treatment occur when a diagnostic test result is positive, particularly in the public sector. However, despite the fact that most public outlets stock anti-malarials (88%), only 61% of people who reported a positive test and were treated exclusively in the public sector reported receiving anti-malarial treatment. Lower levels of treatment of positive cases in the private sector (42%) could be due to less favourable availability of anti-malarials in the private sector. Anti-malarial stock-outs may account for some of the poor case management practices in both the public and private sectors. However, these results suggest that further examination of case management practices in both the private and public sectors is needed to understand prescribing and dispensing behaviour regardless of stocking rates. Interventions that aim to improve fever management and prevent further development of artemisinin-resistant parasites by improving price and availability of effective combination treatments require supporting interventions targeting provider and consumer behaviour and preferences [18]. Interventions to improve appropriate treatment rates such as provider training and supervision are particularly needed in the private sector as its performance lags behind the public sector and it is the source of the majority of cocktail treatments. Approaches currently being explored in Cambodia to improve private provider knowledge and practices include medical detailing [19] and strengthened public-private partnerships [15].

Effective interventions to target the widespread use of cocktail treatments in the private sector will require much more information on what transpires between providers and clients when clients present with "malaria fever." While this study and others conducted to-date document the common practice of cocktail treatment [12,13,16,17], there is need for studies of provider and consumer beliefs and behaviour contributing to these practices. This information can be used to identify key areas for changing knowledge, attitudes and behaviour with respect to cocktail treatment. Research could include qualitative and quantitative studies focused more specifically on the use of cocktails for treating illness more broadly, as opposed to the narrow scope of the studies presented here - focused solely on the anti-malarial market and household treatment of "malaria fever."

### Study limitations and need for additional research

There are a number of limitations to this study, which may be important for interpretation of results regarding the anti-malarial market as well as diagnosis and treatment of suspected malaria. First concerning the anti-malarial market, estimating relative volumes of anti-malarials sold/distributed in the week prior to the survey relies upon converting the amount of each drug reportedly sold/distributed into a comparable unit - the adult equivalent treatment dose (AETD). However, anti-malarials may be sold/distributed in sub-standard doses. The use of AETDs, therefore, systematically underestimates the actual market share of anti-malarials that are sold/distributed in sub-standard doses. This is a concern particularly with respect to oral artemisinin monotherapy, where the AETD is calculated as a seven-day treatment however the amount sold/distributed may often be sub-standard. Additionally, the methodology of calculating AETD is limited when AETD cannot be calculated due to missing information. Missing data that prohibit volumes calculation include missing strength information, which can occur when loose tablets are stored in unlabelled or partially labelled containers. In other cases, information on the volumes sold/distributed is missing. In this study 14% of the N = 2,036 audited anti-malarials were excluded from the volumes calculation due to missing information. Excluded anti-malarials were primarily chloroquine (38%) and quinine (27%), meaning that the relative volumes estimates reported in this study underestimate the contribution of these non-artemisinin monotherapies. Anti-malarials excluded from volumes calculations are primarily from the private sector (80%), particularly from village shops (29%) and other small-scale providers. The relatively high contribution of the private sector to national sales volumes reported in this study is therefore a conservative estimate.

Household survey results rely on respondent recall regarding diagnostic testing, testing results and subsequent treatment. A very high proportion of those who received a diagnostic test reported positive results (88%). The category of respondents in this study who reportedly had a positive test result could be over-inflated and perhaps better represents perceived positive rather than actual positive test results. Another limitation that could have contributed to this high positivity rate is the screening process, which aimed to identify people who had "malaria fever." People who tested negative may have responded "no" to the question on recent "malaria fever," because they received a confirmed negative diagnosis. However, the extent of this exclusion seems limited given that previous studies [13], including the CNM 2007 national household survey focused on fever have also found very high self-reported positivity rates [7]. Gathering unbiased information on diagnostic test results through household surveys is generally a challenge and a barrier to constructing relevant, unbiased treatment outcome indicators where policy dictates treatment based on test results.

One of the key aims of the household survey was to gather information on the consumer side of the anti-malarial market. As such, the household survey focused on self-reported "malaria fevers" because, given relatively low malaria prevalence in Cambodia, studying all fevers would be unlikely to yield sufficient information on anti-malarials consumed. Balancing the need to gather information about anti-malarials acquired and consumed with limited time and money for fieldwork required this focus. To obtain information on just N = 426 anti-malarial treatments acquired by households, over 22,000 households were screened and N = 1,617 fever episodes were studied. In comparison, the 2007 CNM survey focused on treatment-seeking behaviour for fever conducted N = 1,316 interviews and obtained information on just N = 93 anti-malarials [7]. Results from this study are therefore useful in understanding treatment-seeking behaviour among people who suspect that they have recently been infected with malaria, and are not necessarily adequate for understanding actions taken when any fever strikes.

Reported use of cocktails was very common in this study, and most of these cocktails were obtained in the private sector (90%). Respondent reports of cocktail treatments in the household survey could refer to the small plastic bags containing multiple types of drug tablets. However it is possible that respondents also referred to receipt of multiple drugs - including partial and/or full-course treatments from one source - as a cocktail. The latter definition of a cocktail is no doubt represented to some extent in the data, given that 10% of "cocktails" were obtained in the public sector, where presumably small plastic bags containing multiple types of drug tablets are not distributed. Further research is needed to document dispensing practices, particularly in the private sector so as to understand the range of treatments that are defined as "cocktails" by consumers.

The nature of cocktail treatments - a mixture of various unmarked tablets and/or blister packages - makes identification of what exactly was consumed by respondents very difficult. Every effort was taken to improve respondent recall though the use of photographs of common pre-packaged therapies as well as individual anti-malarial tablets. Results suggest that in many cases, people take a cocktail with no identifiable anti-malarial. These results do not necessarily mean that cocktails do not contain anti-malarials, rather that despite rigorous efforts, interviewers and respondents could not identify any anti-malarials in the cocktail. These results are contrary to previous findings that cocktails acquired for malaria in Cambodia more often than not reportedly contained an anti-malarial [13]. There is need for more research to understand the contents of cocktails acquired for "malaria fever," both in the context of a positive diagnostic test results and in the case of no test. While it is possible that these cocktails contain full-course treatments, it is also likely that they do not, which is a concern both for the individual patient and for resistance containment efforts.

Findings from this study show that the majority of providers in both public and private outlets are familiar with the first-line treatment for malaria in Cambodia. The reason that providers sell/distribute cocktails rather than full-courses treatments, including pre-packaged ACT, particularly given reasonable availability and price, is not well understood. From a research perspective, respondent inability to report contents of their cocktail is a limiting factor in understanding treatments acquired and consumed. From a programme perspective, these limitations to respondent recall suggest that there is little communication between providers and clients on cocktail contents and instructions for correct adherence. CNM survey results suggest that there is little demand for full-course treatment; only 39% of household respondents indicated awareness of the need to adhere to the full treatment course [7]. Raising awareness among the general population at risk for malaria and encouraging acquisition of full-course pre-packaged therapies are critical to improving malaria treatment in Cambodia.

## Conclusion

Evidence concerning the availability, price and volumes of anti-malarials moving through the public and private sectors suggests that the anti-malarial market in Cambodia is favourable for both effective fever management and prevention of the spread of artemisinin drug resistance. Sale/distribution of artemisinin monotherapies is still a problem but is relatively low, and sale/distribution of ACT relative to other anti-malarial treatments is high. However, the problem emerging from household survey research is that many "malaria fevers" are not confirmed through diagnostic testing - particularly when treatment is sought in the private sector, and "malaria fever" is most commonly treated with cocktail treatments - many of which do not contain identifiable anti-malarial drugs. There is evidence to suggest that positive diagnostic test results are not consistently treated appropriately, particularly in the private sector. More information is needed to better understand factors that influence provider and client respect for test results. There is urgent need to address the private provider practice of prescribing drug cocktails and consumer preferences for cocktail treatment, particularly given the fact that treatment for "malaria fever" is frequently sought from private providers. Evidence-based interventions that target provider behaviour and aim to increase informed demand among consumers for malaria diagnostic testing and full-course national first-line anti-malarial treatments are needed. These interventions will require additional information to better understand the factors that contribute to cocktail treatment and that serve as barriers to diagnostic testing and appropriate full-course treatments.

## Competing interests

Henrietta Allen is the Malaria Programme Technical Advisor for PSI/Cambodia.

## Authors' contributions

KOC designed the *ACTwatch *Cambodia household and outlet surveys and data collection instruments. DC made contributions to the study design. ML and HG are responsible for the particular analyses presented in this paper. HG and SP made contributions to field work, data cleaning and analyses presented in *ACTwatch *outlet and household study reports. TS, SY and AS assisted with interpretation of findings. All authors read and approved the final manuscript.
